# Brain Tumor Genetic Modification Yields Increased Resistance to Paclitaxel in Physical Confinement

**DOI:** 10.1038/srep26134

**Published:** 2016-05-17

**Authors:** Loan Bui, Alissa Hendricks, Jamie Wright, Cheng-Jen Chuong, Digant Davé, Robert Bachoo, Young-tae Kim

**Affiliations:** 1 Department of Bioengineering, University of Texas at Arlington, TX, USA; 2 Advanced Imaging Research Center, UT Southwestern Medical Center, TX, USA.; 3 Department of Neurology and Neurotherapeutics, UT Southwestern Medical Center, TX, USA; 4 Department of Urology, UT Southwestern Medical Center, TX, USA.

## Abstract

Brain tumor cells remain highly resistant to radiation and chemotherapy, particularly malignant and secondary cancers. In this study, we utilized microchannel devices to examine the effect of a confined environment on the viability and drug resistance of the following brain cancer cell lines: primary cancers (glioblastoma multiforme and neuroblastoma), human brain cancer cell lines (*D54* and *D54-EGFRvIII*), and genetically modified mouse astrocytes (wild type, *p53−/−, p53−/− PTEN−/−, p53−/− Braf*, and *p53−/− PTEN−/− Braf*). We found that loss of *PTEN* combined with *Braf* activation resulted in higher viability in narrow microchannels. In addition, *Braf* conferred increased resistance to the microtubule-stabilizing drug Taxol in narrow confinement. Similarly, survival of *D54-EGFRvIII* cells was unaffected following treatment with Taxol, whereas the viability of *D54* cells was reduced by 75% under these conditions. Taken together, our data suggests key targets for anticancer drugs based on cellular genotypes and their specific survival phenotypes during confined migration.

More than 120 types of primary tumors can occur in the human nervous system, and detrimentally affect the life of many people at all ages^1^,^2^. These effects become more severe in patients with metastatic cancers, which present major challenges in therapeutic treatments. For instance, neuroblastomas, which are thought to form during the development of the peripheral nervous system^3^, have been reported to have an occurrence of 10.9 per million children and 52.6 per million infants anually^4^. Among all patients suffering from neuroblastomas, more than 50% are diagnosed with metastasis^5^. Another example is glioblastoma multiforme (GBM), a grade IV glioma, which has an occurrence of 3.19 per 100,000 people, and represents 16% of all primary brain cancers^6^,^7^. GBM is the most common type of malignant brain cancer, a finding that is exacerbated by its rapid growth and highly diffuse infiltration^6^. Consequently, patients with neuroblastoma or GBM have 5-year survival rates of 59% and 5%, respectively^4^,^7^.

Despite numerous antitumor drugs, cancer cells remain highly resistant to chemotherapy treatment. In many cases, the tumors become highly malignant and develop secondary cancers^8^. It is thought that during infiltration through tissues, lymphatic vessels, white matter tracts, etc., cancer cells encounter a specific degree of physical confinement^9^. Recent studies of invasive cancers have not only elucidated the mechanisms of cellular adaptation in confinement, such as the change in cell morphology and migration modality^10^,^11^,^12^,^13^,^14^ but also emphasized their resistance to many chemotherapeutic drugs^15^. Herein, we report the resulting effect of physical confinement on anticancer drug resistance of different cancer cell lines, including those with different stages of carcinogenic mutations.

We used engineered Polydimethylsiloxane (PDMS) microfluidic devices to study the effects of Paclitaxel (referred to as Taxol) on primary cancers and genetically modified cell lines in three different physical confinements. Taxol was chosen as a model anticancer drug due to its known efficacy in the treatment of many cancers, given its effects on microtubule assembly, which results in the apoptosis of tumors^16^,^17^. The micro-environment for this study, depicted in Fig. 1, consisted of three different degrees of physical confinement, as follows: narrow confinement (5 by 5 μm in height and width, denoted as 5_5) for observing the cells when they were forced to alter their structure to migrate; wide confinement (15 by 15 μm in height and width, denoted as 15_15), where cells could maintain their structure while still being confined in a 3D space; and the 150 μm high reservoir (denoted as 2D), which simulated the environment of traditional cell culture plates.

For the first part of this study, we evaluated the effects of both physical confinement and Taxol administration on the primary cancers of the nervous system, GBM and neuroblastoma. We found higher viability in untreated cells (i.e., cells maintained in Taxol-free medium) than in Taxol-treated cells, and when in a higher degree of physical confinement, the treated cells appear to be more tolerant to the toxic effect of Taxol. For the second part, with a similar setup, we examined the effect of physical confinement on the viability of cell lines that had different combinations of mutant genes. We first used human brain cancer *D54* and *D54-EGFRvIII* cell lines, in which *D54-EGFRvIII* was more malignant due to the constitutive activation of mutated epidermal growth factor receptor (EGFR) associated with the overexpression of wild-type EGFR (wt EGFR)^18^,^19^,^20^. Next, we studied the effects of physical confinement and Taxol administration on mouse astrocytes with different levels of cancerous mutations. The cell lines studied included wild-type and four genetically modified mouse astrocyte lines with different levels of cancerous mutations, including single tumor suppressor gene mutation (*p53−/−*), double tumor suppressor gene mutations (*p53−/− PTEN−/−)*, single tumor suppressor gene with oncogene mutation (*p53−/−Braf*), and double tumor suppressor with additional oncogene mutations (*p53−/− PTEN−/− Braf*). The level of cancerous mutations in *p53−/− PTEN−/− Braf* was considered to be the highest and resembled highly malignant brain cancer, whereas the others, *p53−/− PTEN−/−, p53−/− Braf*, and *p53−/−,* represent the middle and low cancerous tumor cells. In our last experiment, we used another antitumor drug with a different mechanism from that of Taxol to test the drug resistance of *D54, D54-EGFRvIII*, and GBM in physical confinements. We used Doxorubicin (Dox) as the drug model, given that unlike Taxol, which targets microtubule, Dox is toxic mainly for its effects on the DNA of cancer cells^21^,^22^,^23^,^24^.

## Results

### Two primary brain cancer cell lines showed high resistance to Paclitaxel in physical confinement

Physical confinement has been shown to affect a number of cellular phenotypes, such as cell morphology and migration motility^25^,^26^. Herein, we found that physical confinement results in a considerable increase in the cell’s resistance to anticancer drug treatment, as revealed in their higher viability when all other factors were kept the same.

Untreated GBM (i.e., GBM in Taxol-free medium) survived in all three physical confinements equally. The viabilities were approximately the same, at 0.84(±0.11), 0.81(±0.14) and 0.82(±0.15) for cells in 5_5, 15_15 and 2D, respectively (Fig. 2a, red line). With Taxol treatment, we found a significant reduction in the viability of GBM cells in the 15_15 and 2D environments, at 0.18(±0.13) and 0.02(±0.04), respectively, with P < 0.01 when compared with untreated cells. However, the Taxol-treated cells in 5_5 microchannels maintained a relatively high viability, 0.72(±0.16) (Fig. 2a, blue line). Statistical analyses revealed significant differences between the viability of Taxol-treated cells in the 5_5 microchannels and those in either the 15_15 microchannels or on a 2D surface.

Neuroblastoma cells showed a different pattern of Taxol resistance and resulting viability in three different physical confinements. There was a slight decrease in the viability of untreated cells in the 5_5 microchannels, 0.76(±0.08), compared with those in the 15_15 microchannels and on the 2D surface, 0.95(±0.06) and 0.94(±0.08), respectively. For Taxol-treated cells, while nearly all cells did not survive on the 2D surface, the viability of cells in the 5_5 and 15_15 microchannels only decreased 28% (0.76 to 0.55) and 18% (0.95 to 0.78), respectively, compared with untreated cells in the same physical environment (Fig. 2b).

### Viability of Taxol-treated D54-EGFRvIII was higher than that of D54 in physical confinement

To understand how genetic mutations affect the viability of cancer cells in physical confinement, we performed another study with two glioma brain tumor cell lines, *D54* and *D54-EGFRvIII*, in which *D54-EGFRvIII* was genetically modified *D54* in the presence of both mutated and overexpressed wild-type (wt) *EGFR*.

Untreated *D54* showed no significant change in viability within any of the three physical environments. Survival ratios included 0.76(±0.26), 0.66(±0.25) and 0.76(±0.14) for cells in the 5_5 and 15_15 microchannels and 2D surface, respectively (Fig. 3a, red line). Taxol-treated cells in all three environments experienced significantly reduced viability from those grown in untreated conditions by 75% (0.76 to 0.19), 92% (0.66 to 0.05) and 100% (0.76 to 0.0), with a significantly higher viability in 5_5 (Fig. 3a, blue line).

Without exposure to the drug administered, *D54-EGFRvIII* showed a significant sensitivity to narrow confinement. The viability of cells in 2D and 15_15 were found to be relatively high, 0.94(±0.07) and 0.88(±0.10), but the viability significantly decreased to 0.69(±0.17) in the 5_5 microchannels (Fig. 3b, red line). When treated with Taxol in 2D, similar to *D54*, almost no *D54-EGFRvIII* cells survived (viability reduced from 0.94 to 0.00). However, the effects of Taxol on *D54-EGFRvIII* in both narrow and wide microchannels were not as profound as those on *D54* cells. The percent decreases in the viability of Taxol-treated cells compared with untreated cells in the 5_5 and 15_15 microchannels were found to be −3% (0.69 to 0.7) and 53% (0.88 to 0.41), respectively (Fig. 3b, blue line). Thus, *D54-EGFRvIII* acquired increased resistance to Taxol in the 15_15 microchannels and even higher resistance in the 5_5 microchannels.

### Loss of PTEN combined with Braf activation resulted in increased viability in physical confinement

We further investigated how genetic mutations in brain tumor cells affected their viability in different physical environments with or without the administration of Taxol. We used wild-type and four genetically modified mouse astrocytes with different levels of genetic mutations, as follows: *p53−/−* (low level), *p53−/− PTEN−/−* (middle level), *p53−/− Braf* (middle level), and *p53−/− PTEN−/− Braf* (high level).

In the wild-type, the viability of untreated cells in 5_5 microchannels decreased significantly by approximately 30% (0.62 versus 0.89 and 0.88) compared with cells grown in both the 15_15 microchannels and on 2D surfaces (Fig. 4a, red line). Taxol-treated cells generally resulted in decreased viability compared with the untreated in the same condition; the viability of Taxol-treated cells in 5_5, 15_15 and 2D were 0.19(±0.20), 0.42(±0.23) and 0.18(±0.18), respectively (Fig. 4a, blue line).

*p53−/−, p53−/− PTEN−/−,* and *p53−/− Braf* showed relatively similar viability patterns to each other. Compared with the same cell line in 15_15 and 2D, untreated *p53−/−* in 5_5 decreased 15% (0.51 versus 0.6) and 42% (0.51 versus 0.87), the *p53−/− PTEN−/−* decreased 26% (0.44 versus 0.6) and 52% (0.44 versus 0.93), and *p53−/− Braf* decreased 29% (0.54 versus 0.76) and 43% (0.54 versus 0.95), respectively (Fig. 4b–d, red line). Taxol-treated cells in 5_5 showed a significantly higher viability than 2D (for both *p53−/−* and *p53−/− PTEN−/−*) and 15_15 (for *p53−/−* alone) (Fig. 4b,c, blue line).

Untreated *p53−/− PTEN−/− Braf* astrocytes survived in all three physical environments equally, showing no significant difference among the viability of cells in 5_5, 15_15 and 2D, 0.92(±0.11), 0.96(±0.06) and 0.87(±0.11), respectively (Fig. 4e, red line). Taxol-treated cells in 5_5, 15_15 and 2D resulted in different levels of decreased viability compared with the untreated cells; the percent decreases, respectively, were 24% (0.92 to 0.7), 28% (0.96 to 0.69) and 93% (0.87 to 0.06) (Fig. 4e, blue line). Moreover, the Taxol-treated cells in both 5_5 and 15_15 microchannels obtained significantly higher viability compared with those in 2D (0.70 and 0.69 versus 0.06) (Fig. 4e). In addition, the absolute viabilities of Taxol-treated *p53−/− PTEN−/− Braf* in 5_5 and 15_15, 0.70(±0.21) and 0.69(±0.23), were much higher than those of the other cell lines: *p53−/−* with 0.32(±0.16) and 0.11(±0.20), *p53−/− PTEN−/−* with 0.30(±0.19) and 0.16(±0.13), *p53−/− Braf* with 0.24(±026) and 0.21(±0.25), respectively (Fig. 4b–e, blue line).

### Taxol diffusion completely and quickly saturated the entire cell in different confined spaces

To ensure that the observed difference in viability was not affected by the differences in cellular morphometry in different confined spaces, we developed a model to evaluate the effective diffusion resistance and the time to saturate the cell to the desired concentration of Taxol (Fig. 5). The differences in cellular morphometries at three physical confinements that could affect the effective diffusion resistance of the drug were mainly based on their available membrane area for drug uptake and diffusive path (*∆x*). Table 1 summarized the *∆x* and calculated values of available membrane area for cells in three confinement cases. For a cell confined in the 5_5 microchannel, the effective diffusion resistance to the drug influx was 56 times higher than that in 15_15 and on the 2D surface. These findings also suggested that the diffusional flow rate of Taxol influx into the cell is 56 times lower in 5_5 than in the other two cases.

Moreover, these results showed that even at a low diffusivity (*D*_*cyto*_ = *1% D*_*H2O*_), because of the small dimension of the cell, the Taxol diffusive influx was complete and saturated the entire cell quickly in less than 2 minutes for all three cases, despite the much higher diffusion resistance of the 5_5 when compared with the other two cases (Table 1). Note that in the calculation, the same ratios (~56:1) were applied to the time rates of Taxol uptake, where the differences are most pronounced during the early stage of the diffusion process. However, whether the much slower time rate of Taxol intake with the resident cell in 5_5 and the actual rate of binding/reaction kinetics contributed to the observed variability, in addition to the sensitivity to specific mutations, remains a question that requires further study.

### Doxorubicin resulted in lower drug resistance in physical confinement compared with Taxol

The drug resistance of confined cells was further investigated using Doxorubicin (Dox) as another antitumor agent, given that Dox kills cancer cells by damaging cellular DNA^21^,^22^,^23^,^24^. We observed that Dox-treated *D54-EGFRvIII* resulted in higher viability in both narrow and wide confinement compared with *D54* (Fig. 6a,b, green line). Dox-treated *D54* and *D54-EGFRvIII* in 5_5 microchannels also showed significantly higher viability than cells in 2D, 0.17(±0.23) versus 0.01(±0.02) and 0.37(±0.31) versus 0.01(±0.03), respectively. Unlike the Taxol case, the viability of Dox-treated *D54-EGFRvIII* in 5_5 microchannels was no longer close to the untreated cells. Moreover, the viabilities of Dox-treated cells were significantly lower than Taxol cases in nearly all confinements (Fig. 6a–c). Experiments with Dox-treated GBM further showed significant cell death, and there was no significant difference of cellular viability among the three physical confinements (Fig. 6c, green line). When the Dox concentration was reduced lower than 1 μM (500 nM and 100 nM), the overall viability of GBM increased. Interestingly, for each Dox concentration, we also found no significant difference in the viabilities among cells in 5_5, 15_15 and 2D, suggesting their insensitivity to the differences in the three physical confinements (Supplementary Figure S2).

## Discussion

Our results clearly showed two major factors that could directly affect brain cancer cell viability: 1) the degrees of physical confinement and 2) the levels of genetic mutation of tumor cells. For all untreated cell lines, given that the medians of viability decreased when the degree of physical confinement became higher (Fig. 7a), the cells apparently encountered a higher level of physical challenge within the narrow microchannels compared with both the wide microchannels and 2D environment. Furthermore, the increased ranges of viability in the narrower confinement indicated varying responses to such a challenge among different cell lines. The two cell lines achieving the highest viabilities in 5_5 microchannels were *p53−/− PTEN−/− Braf* astrocytes and GBM while the two lowest viabilities were *p53−/−* and *p53−/− PTEN−/−* astrocytes. In addition, the viabilities of *p53−/−* and *p53−/−Braf* astrocytes in 5_5 microchannels were approximately the same (0.51 versus 0.54). These results also revealed that the low- and middle-level mutation did not provide the astrocytes with the ability to survive in a narrow confinement as remarkably as the high-level mutated astrocytes and grade IV malignant brain cancer GBM. Both *p53* and *PTEN* are tumor suppressors that are known to regulate cellular proliferation and apoptosis^27^,^28^. Mutations of *p53* and *PTEN* are commonly found in brain tumors; antisense *PTEN* also affects tumor invasion and metastasis via the down-regulation of the interaction between cells and the extracellular matrix^28^,^29^,^30^. However, if the deletion of these genes is more related to uncontrolled growth and decreased cellular adhesion that can only increase cell migration to a specific degree, *p53−/−* and *p53−/− PTEN−/−* astrocytes might not be capable of withstanding the narrow confinement. *Braf* is known as an oncogene that functions through a proliferation-inducing pathway (e.g., the MEK pathway)^31^,^32^. It was found to overexpress in various aggressive and invasive tumors, and exhibits a particularly higher expression in metastatic cells than primary tumors^33^. Moreover, *Braf* mutations occur as the level of severity increases, causing the existing cancer to become more cancerous^34^,^35^. In our study, the presence of *Braf* with *p53−/−* alone (middle level mutation) did not result in increasing cellular viability in the confinements compared with *p53−/−* and *p53−/− PTEN−/−*, whereas cells with triple-mutant *p53−/− PTEN−/− Braf* (high level mutation) could survive in narrow confinement without any difficulty (Fig. 4e). Our results revealed the role of the oncogene *Braf* on inducing lower level mutated cells to adapt to a narrow physical confinement. These findings also helped to explain why *Braf* mutation increases cellular invasive migration, inducing the tumor to demonstrate higher levels of malignancy.

The effect of Taxol in cancer treatment is well understood^16^,^17^,^36^,^37^. With its major function to stabilize microtubules, Taxol is most effective when targeting fast-dividing cells that employ the microtubule-based spindle apparatus^16^,^36^. This explains why the effect of Taxol is dramatic when cells are grown in a 2D environment. Excluding wild-type astrocytes, the viability of all fast-dividing cells is within the range of 0.00 to 0.06. At increasing levels of physical confinement, the percentage of cell death also decreased (Fig. 7b–d). We have illustrated that the cellular uptake of Taxol by diffusion complete with drug molecules saturated the cells quickly despite the changes in morphology and increasing cellular effective diffusion resistance to drug influx in narrow microchannels. Furthermore, we illustrated the expected Taxol concentrations at the cell boundaries within the microchannel using computer modeling (Supplementary Figure S1). Thus, any change in viability could be directly related to the response to the drug in particular Taxol-treated cells. This finding also indicated that the cells acquired specific resistance to Taxol in the narrow confinement. Several studies showed that during active migration, cancer cells ceased to divide^38^; in addition, the cellular phenotype transforms to support the invasion process^39^,^40^. Moreover, genetic modification that increases the malignancy of cells resulted in a higher resistance to the drug when in confinement. Indeed, in the 5_5 microchannels, the normalized viability of *D54-EGFRvIII* was approximately 1.00 compared with that of *D54* (0.25) (Fig. 7c); *p53−/− PTEN−/− Braf* achieved 0.76 normalized viability compared with that of *p53−/−, p53−/− PTEN−/−*, and *p53−/− Braf*, which were 0.67, 0.63, and 0.45, respectively (Fig. 7d). Our findings provide a means to identify which mutations during cancer development play major roles in enhancing cellular survival in confinement and also show the relationship of such genetic alterations to the resistance against Taxol when the cells migrate.

Chemotherapeutic drugs targeting microtubules might not be sufficient to completely stop the progression of malignant cancers. Instead, a thorough understanding of the mechanisms and more efficient treatments of high-grade cancers should be further investigated. For instance, 20–30% of human glioblastomas are related to the *EGFRvIII* mutation^41^, and the cells that contain this mutation show increased levels of invasion and viability^42^,^43^. Montgomery *et al*. showed that the *EGFRvIII* mutation modulated tubulin expression, thus increasing Taxol resistance^44^. Moreover, other studies suggested immune-targeting *EGFRvIII* in the inhibition of brain tumor infiltration^45^,^46^,^47^. Similarly, the use of *Braf* inhibitors, such as Dabrafenib and other *MEK* inhibitors, have also shown their effectiveness in clinical trials of melanoma and brain metastases^48^,^49^,^50^. By focusing on drugs that target malignant mutations, we showed the efficacy of our device in the analysis of brain cancers.

Our microfluidic system design is novel because it provides a platform offering different channel dimensions with varying degrees of physical confinement for seeded cells in the central reservoir to migrate on their own. It could be used to test and evaluate the effect of different drugs on cancer cell migratory phenotypes; it could also be used to investigate the mechanisms of drug resistance of cancer cells in physical confinement. In our last experiment, Doxorubicin (Dox) significantly reduced cellular viability more than Taxol. Moreover, Dox killed cancer cells regardless of the degree of physical confinement, a finding that has been consistently observed within a broad range of Dox concentrations. This result suggests that microtubule targeting drugs such as Taxol are selectively sensitive to confinement and becomes ineffective in killing migrating cells. However, the use of Dox, which targets cellular DNA, increases the toxic effect and presents a better candidate for use as a chemotherapy for metastatic cancers^51^.

## Conclusions

Genetic mutations strongly promoted cell survival in physical confinement and in the tolerance to Taxol; cells with high levels of carcinogenic mutations showed significantly higher levels of viability similar to those observed in primary cancer cells. Results from our study also help to explain the survival of many cancer cells in the later stages of cancer, and they also provide insight toward why many anticancer drugs fail to cease metastasis. The microfluidic system developed by our group allows the continuous monitoring of cellular behaviors in different physical confinements. It also presents a novel *in vitro* validation methodology and platform for anti-metastatic drug testing.

## Methods

### Fabrication of PDMS microfluidic devices

**Figure 1:**
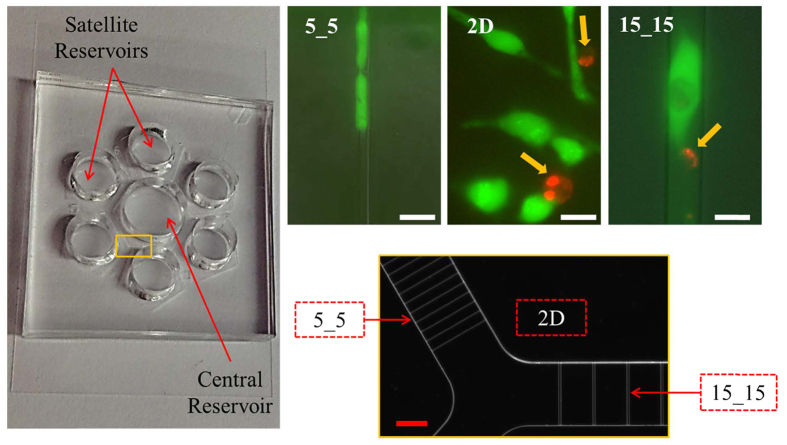
Experimental setup for studying the anticancer drug response in different physically confined and non-confined environments. The PDMS microfluidic device (left) and zoom-in microchannels (bottom right). The microchannels are areas within the doublet lines. The three areas of interest include the reservoir with a 150 μm height (2D environment) and the 5_5 and 15_15 microchannels. Examples of living (green) and dead cells (red, yellow arrows) located in the three different physical environments (top right). White scale bar: 15 μm. Red scale bar: 200 μm.

**Figure 2:**
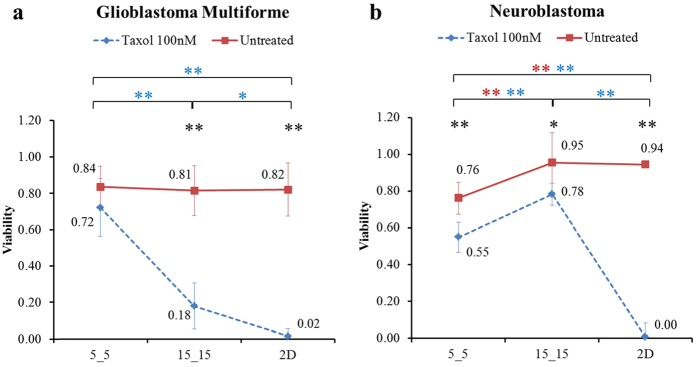
Increase of Taxol resistance of two primary brain cancer cell lines within physical confinement compared with cells grown on 2D. Effect of physical confinement and Taxol on the viability of (**a**) GBM and (**b**) neuroblastoma. Average ± standard deviation used in the analyses. *P < 0.05, **P < 0.01 comparison of the viability between untreated and Taxol-treated cells. *P < 0.05, **P < 0.01 comparison of the viability among three different physical environments of the untreated cells. *P < 0.05, **P < 0.01 comparison of the viability among three different physical environments of Taxol-treated cells.

**Figure 3:**
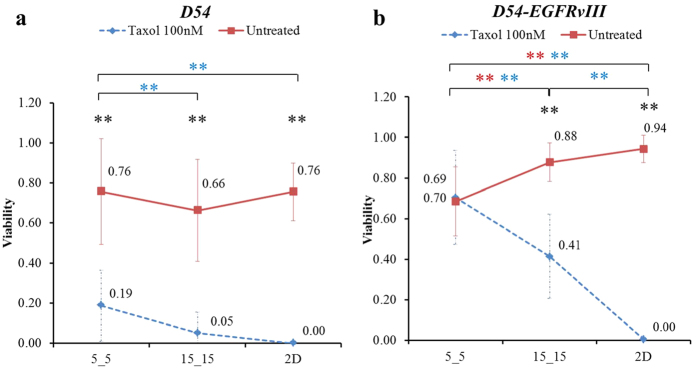
Viability of Taxol-treated *D54-EGFRvIII* was higher than that of *D54* in physical confinement. Effect of physical confinement and Taxol on the viability of (**a**) *D54* and (**b**) *D54-EGFRvIII*. Average ± standard deviation used in analyses. **P < 0.01 comparison of the viability between untreated and Taxol-treated cells. **P < 0.01 comparison of the viability among three different physical environments of the untreated cells. **P < 0.01 comparison of the viability among three different physical environments of Taxol-treated cells.

**Figure 4:**
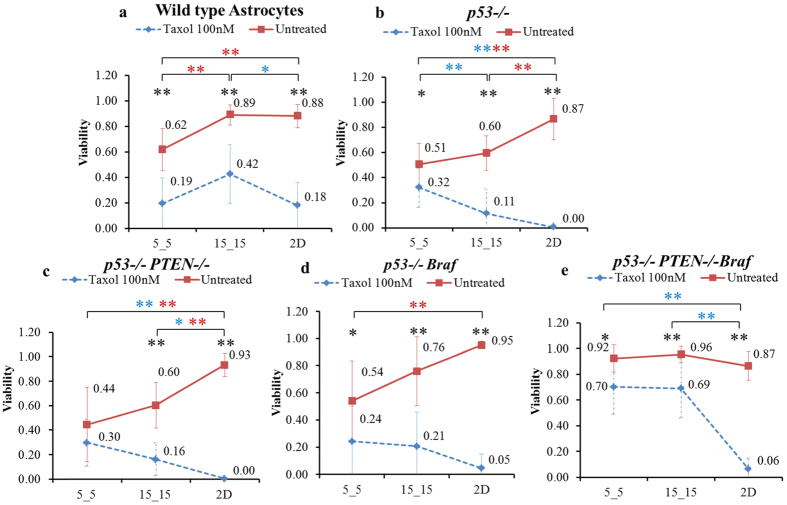
Loss of *PTEN* combined with *Braf* activation resulted in increased viability in physical confinement. Effect of physical confinement and Taxol on the viability of (**a**) wild-type mouse astrocytes and four genetically different mouse astrocytes, which included (**b**) *p53−/−*, (**c**) *p53−/− PTEN−/−*, (**d**) *p53−/− Braf,* and (**e**) *p53−/− PTEN−/− Braf*. Average ± standard deviation used in the analyses. *P < 0.05, **P < 0.01 comparison of the viability between untreated and Taxol-treated cells. *P < 0.05, **P < 0.01 comparison of the viability among three different physical environments of the untreated cells. *P < 0.05, **P < 0.01 comparison of viability among three different physical environments of Taxol-treated cells.

PDMS (Dow Corning, Sylgard 184) microfluidic channels were fabricated using standard photo- and soft-lithography techniques^52^,^53^, which endowed the device with three distinct features, as follows: narrow confinement (5 by 5 μm in height and width, denoted as 5_5), wide confinement (15 by 15 μm in height and width, denoted as 15_15), and a 150 μm high reservoir (denoted as 2D) (Fig. 1). All microchannels were 530 μm in length.

Once the PDMS was solidified, holes were punched to create a large central well (8 mm in diameter) and 6 satellite wells (6 mm in diameter), which served as inlets for seeding cells and supplying the cells with proper nutrition. The sterilized PDMS devices were placed onto a cover glass to create confined microchannels and were then subsequently coated with 10 μg/ml laminin (Sigma-Aldrich) prior to the introduction of cells.

### Cell lines and culture

All cell lines were provided by the University of Texas Southwestern Medical Center at Dallas with the approval of IRB. Patient-derived GBM (*Ink−/−, CD133+, EGFRvIII*)^26^,^54^,^55^,^56^,^57^ and Neuroblastoma (SH-SY5Y cell line human) were used as two primary cancer models. Different genetically modified cells, including two human brain tumor cell lines (*D54,* human astrocytoma cell line derived from a patient with glioblastoma multiforme (grade IV) and *D54-EGFRvIII*) and four different gene-altered mouse astrocytes mimicking different levels of human cancerous cells (*p53−/−, p53−/− PTEN−/−, p53−/− Braf*, and *p53−/− PTEN−/− Braf* astrocytes) were used. These four types of mouse astrocytes were derived from the same wild-type mouse astrocytes.

The cells were seeded in the central reservoirs of the devices (Fig. 1). GBM cells were maintained in serum-free Dulbecco’s Modified Eagle’s Medium/F-12 medium (DMEM/F-12) supplemented with 2% B-27 (Invitrogen), 0.25% Insulin-transferrin-selenium-X (Invitrogen), 25 μg/ml gentamicin, 20 ng/ml human bFGF (basic fibroblast growth factor), and 20 ng/ml mouse EGF (epidermal growth factor). For the other cells, we used DMEM/F-12 medium with 10% fetal bovine serum.

### Quantitative comparison of viability in three different physical environments

Once there were a sufficient number of cells that migrated into the microchannels, untreated cells were continually maintained in the drug-free medium while drug-treated cells were incubated in fresh medium in the presence of the drug. We used 100 nM Taxol (Paclitaxel, Sigma-Aldrich) for 72 hours and 1 μM Dox (Doxorubicin, Sigma-Aldrich) for 48 hours. This condition resulted in the death of approximately more than 80% of the cells on conventional 2D culture plates (Supplementary Figure 2) allowing us to investigate any increase in viability in more confined environments. The cell viability was determined using Propidium Iodine (MP Biomedicals)/Fluorescein Diacetate (Sigma-Aldrich) (PI/FDA) staining. In terms of cell viability, we quantitatively compared the effects of physical confinement (5_5, 15_15 and 2D) and Taxol administration for different cell lines. The absolute viability was calculated based on the ratio of living cells. The normalized viability was calculated by dividing the absolute viability of the Taxol-treated cells by the absolute viability of the untreated cells in the same condition.

### Evaluation of effective diffusion resistance and drug saturation

**Figure 5:**
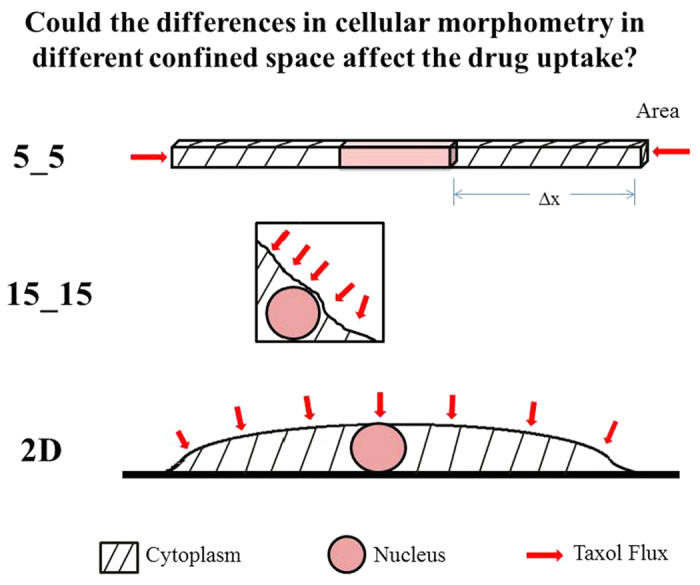
Illustrations show differences in cellular morphometry in different confinement environments (5_5, 15_15, and 2D), where 5_5 is shown in a perspective view and both 15_15 and 2D are shown in a cross-sectional view. Shaded regions represent the cytoplasm; wine-colored regions indicate the nucleus; and the red-arrowheads depict Taxol influx into the cell through the available membrane area.

**Figure 6:**
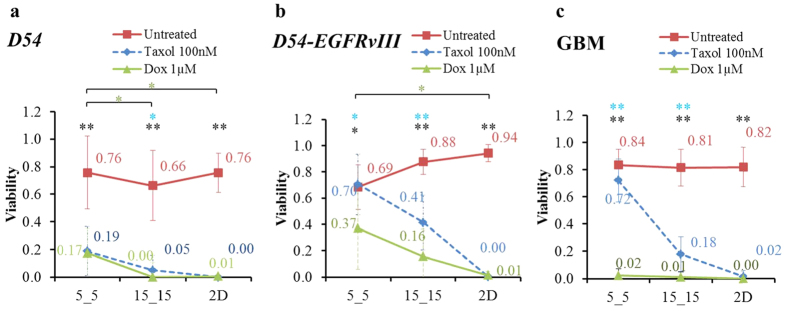
Lower viability of Dox-treated brain tumor cell lines (**a**) *D54,* (**b**) *D54EGFRvIII,* and (**c**) human GBM compared with Taxol-treated cells. *P < 0.05, **P < 0.01 comparison of viability between control (untreated) and Dox-treated cells. *P < 0.05 comparison of viability among three different physical environments of the Dox-treated cells. *P < 0.05, **P < 0.01 comparison of viability between Taxol- and Dox-treated cells.

**Figure 7:**
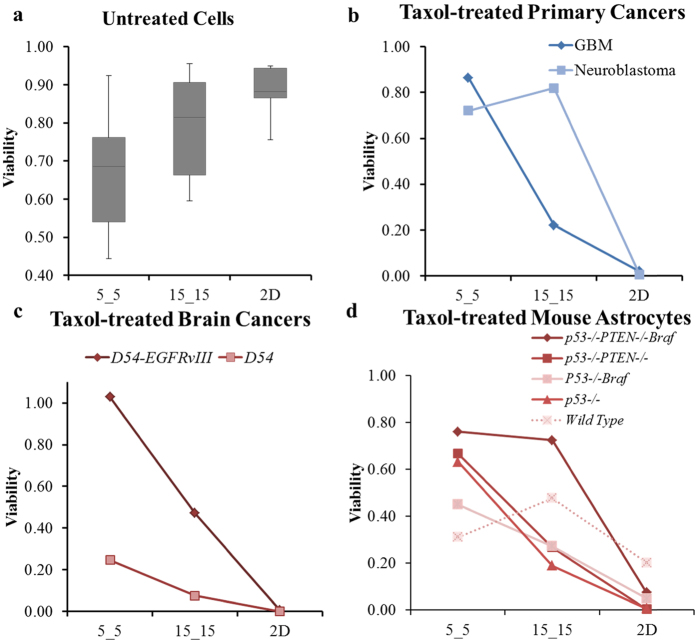
Degree of survival and resistance to anticancer drug in physical confinement was different among cell lines. (**a**) Viability distribution of all untreated cells in three different physical environments. The horizontal lines represent the medians, and the upper and lower whiskers indicate the highest and lowest values. Normalized viability of Taxol-treated primary cancers (**b**), two brain cancer cell lines (**c**), and different genetic mouse astrocytes (**d**).

Next, we asked whether the differences in cellular morphometry in different confined spaces (Fig. 1) could affect the drug uptake and contribute to the observed differences in cellular viability (Fig. 5). To assess this phenomenon, we used a simple one degree of freedom model. We expressed the effective diffusion resistance to drug influx as the ratio of driving concentration differences *(∆C)* over the rate of drug uptake (i.e., diffusional flow rate), where the resistance is a function of the drug diffusivity in cytoplasm (*D*_*cyto*_), the available membrane area (*Area*), and the length of diffusive path (*∆x*), written as 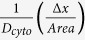. That is, for a fixed diffusivity *D*_*cyto*_, the effective diffusion resistance is proportional to *∆x*, inversely proportional to the available membrane area, as shown in Eq. 1:


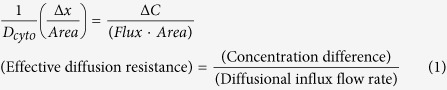


For a typical cell (with a volume of 1,250 μm^3^) in a confined 5_5 microchannel, the membrane area available for drug uptake is 25 μm^2^ at each end, resulting in a total of 50 μm^2^. The membrane area available for drug uptake is estimated to be 280 μm^2^ when it is on a 2D surface, and ~250 μm^2^ in a 15_15 microchannel where the tumor cell adheres to both the bottom and side surfaces of the microchannel. For a cell in the 5_5 microchannel, the diffusive path length *∆x* is estimated to be 20 μm from the entry plasma membrane to the nucleus, whereas *∆x* is estimated to be 2 μm for both the 2D and 15_15 environments given that the nucleus is within the short diffusive distance from the plasma membrane. Because no data are available for Taxol diffusivity in the cytoplasm of tumor cells examined, we expressed *D*_*cyto*_ as a fraction of *D*_*H2O*_ (*D*_*cyto*_ = 10%, 5%, or 1% of *D*_*H2O*_) and calculated the time that it takes to saturate a cell to 100 nM Taxol when grown in the three different types of environments.

### Statistical Analyses

All data were presented as the average ± standard deviation (total cells n ≥ 30 for each condition). For each condition, a paired t-test was performed to compare the viability of the untreated and drug-treated cells. One-way ANOVAs were performed for comparison of the significance among multiple groups. When the P-values were significant, Tukey post-hoc tests were performed to identify the difference between the groups.

## Additional Information

**How to cite this article**: Bui, L. *et al*. Brain Tumor Genetic Modification Yields Increased Resistance to Paclitaxel in Physical Confinement. *Sci. Rep.* **6**, 26134; doi: 10.1038/srep26134 (2016).

## Supplementary Material

Supplementary Information

## Figures and Tables

**Table 1:** A summary of values for the available membrane area and the length of diffusive path for three confinement cases along with the calculated time to saturate a cell to 100 nM for different assumed values in Taxol diffusivity in cytoplasm (*D*_*cyto*_).

**Channel/ Geometry**	**Cell Volume (μm**^**3**^)	**Available Membrane Area for Drug Uptake (μm**^**2**^)	**Diffusive Path** ***∆x*****(μm)**	***∆x*****/Area (μm**^**−1**^)	**Ratio in Effective Diffusion Resistance**	**Time to Saturate the Cell to 100** nM **(sec)**		
						**if** ***D***_***cyto***_** = 10% of** ***D***_***H2O***_	**if** ***D***_***cyto***_** = 5% of** ***D***_***H2O***_	**if** ***D***_***cyto***_** = 1% of** ***D***_***H2O***_
5_5	1,250	50	20	0.4	56	11	22	110
15_15	1,250	250	2	0.008	1.12	0.22	0.44	2.2
2D	1,250	280	2	0.00714	1	0.2	0.4	2.0

A value of *D*_*H20*_ = 430 μm^2^/sec was used^58^ as the reference.
