# Supplementary material for: Brain Tumor Genetic Modification Yields Increased Resistance to Paclitaxel in Physical Confinement
Source: Sci Rep. 2016 May 17;6:26134. doi: 10.1038/srep26134 (PMC4869028; doi:10.1038/srep26134)
Supplement: Supplementary Information [file srep26134-s1.pdf]

Brain Tumor Genetic Modification Yields Increased Resistance to Paclitaxel in Physical Confinement

Loan Bui[1], Alissa Hendricks[1], Jamie Wright[1], Cheng-Jen Chuong[1], Digant Dave[1], Robert Bachoo[2], and Young-tae Kim[1, 3]*

[1]Department of Bioengineering, University of Texas at Arlington, TX

[2]Department of Neurology and Neurotherapeutics, UT Southwestern Medical Center, TX

[3]Department of Urology, UT Southwestern Medical Center, TX

Supplementary Information

*Corresponding author:

Young-tae Kim, Ph.D.
Department of Bioengineering
500 UTA blvd ERB244
University of Texas at Arlington
Arlington, TX 76010

E-mail: ykim@uta.edu
Fax: 817-272-2251
Phone: 817-272-5023

Supplementary Methods

Drug availability

To predict the spatiotemporal Taxol distribution within a single 5_5 microchannel after the initial introduction of 100 nM Taxol to both the central and satellite reservoirs, we performed numerical simulations (COMSOL 4.4) for the following two cases: 1) media in the microchannel was free from Taxol and 2) two cells were conservatively positioned, each flushed against either end of the microchannel. In Case 2, cells were assumed to deform to 50 μm axially and fully obstruct the microchannel. The cell dimensions were consistent with the experimental observations of cancer cells under similar conditions of confinement. Taxol transport within the fluid-filled microchannels was taken to be a passive diffusive process governed by Fick's 2nd law (Eq. S1), where *C* refers to the Taxol concentration, *t* is the time elapsed since drug introduction, and *D* is the diffusivity of Taxol in the media or in the blocking cells. For Case 1 simulation, a value of 430 $\mu m^2/sec$[53] was used for the media in the microchannel that is free from Taxol with $D = D_{H2O}$. For Case 2 simulations, D_{cyto} was used in place of *D* for the blocking cells, whereas D_{H2O} was used for the center part of the microchannel between two clocking cells:

$$\frac{\partial C}{\partial t} = D\nabla^2 C \qquad \text{[Eq. Sl]}$$

The microchannel side walls were assumed to be impermeable to drug molecules. Case 2 simulation was used to help assess how cells positioned at the two ends of a microchannel could impede and affect the Taxol diffusion from the reservoirs into the media in the center of the microchannel. The overall resistance from a sitting cell to Taxol diffusion consists of that from the plasma membrane, cytoplasm, nucleus envelope and nuclear contents. No data exist that

accounts for the resistance from each of these components and how they are connected to impede the Taxol diffusion in the cell. For this assessment, we chose to perform a parametric study by assuming the Taxol diffusivity D_{cyto} in a sitting tumor cell to be either 10%, 5% or 1% that of Taxol diffusivity in water D_{H2O}.

Doxorubicin dose response

We examined the dose response of cells cultured on a 2D surface using different Dox concentrations. The cells (*D54*, *D54EGFRvIII* and GBM) were treated with a range of Dox concentrations (0, 1 nM, 10 nM, 100 nM, 500 nM, and 1 μM). The viability of each cell line was determined after 48 hours (n=4/dox concentration). The viabilities at different concentrations were normalized against untreated cells (Dox 0 nM).

Effect of low concentration Dox on the viability of GBM in physical confinements

GBM cells cultured in the devices were treated with 100 nM and 500 nM Dox for 48 hours. The viabilities of cells in different physical confinements (5_5, 15_15, and 2D) were quantified.

Supplementary Results

Virtual confirmation of the availability of Taxol to confined cells within the microfluidic device

Simulation results indicated that Taxol diffusion in the unblocked microchannels resulted in rapid saturation. Volumetric concentration contours (Fig. S1A) illustrated noticeable concentration gradients after one minute as indicated by the color variations. After five minutes, the spatial concentration gradients had disappeared and the concentration at the center of the microchannel had increased to nearly 90 nM. The contours illustrated that the concentration changes were dominant in the axial direction with little transverse variation. Figure S1B showed

the minute-by-minute axial concentration profiles over the first ten minutes of simulation. Clearly, concentration gradients disappeared in the microchannel within 10 minutes of drug introduction.

The effect of confined cells at the microchannel ends is illustrated in Figures S1C-S1E for cases where D_{cyto} = 10%, 5%, or 1% that of D_{H2O}. The existence of steep concentration gradients in the 50 μm regions near both junctions (X = 0 μm and X = 530 μm) reaffirmed that resistances were much higher in the cells than in the microchannels. Diffusion was limited through the cells but drug concentrations equilibrated quickly within the microchannel interior as indicated by the smaller concentration differentials there. With assumed lower Taxol diffusivity, 10%, 5% and 1% that of value in water, the concentration gradients in the cells became increasingly steep and the drug concentration at the microchannel center continued to decrease for a given time. Target concentrations of drug (100 nM) were easily achieved through the center part of the entire microchannel within 3 hours if the Taxol diffusivity in the cell becomes 1%.

Overall, the steady state, uniform concentrations of nearly 100 nM were achieved in a couple of hours. The result indicated that even the coefficient efficiency decreased by 95%, and the desired Taxol concentration should be ready at any microchannels within one hour. Regardless of the higher number of cells inside the microchannel as well as the uptake process of Taxol by cells, the simulation results virtually confirmed that experimental Taxol doses were achieved at cell boundaries after short time windows in the microfluidic device and the experimental results should reflect the appropriate cell exposure.

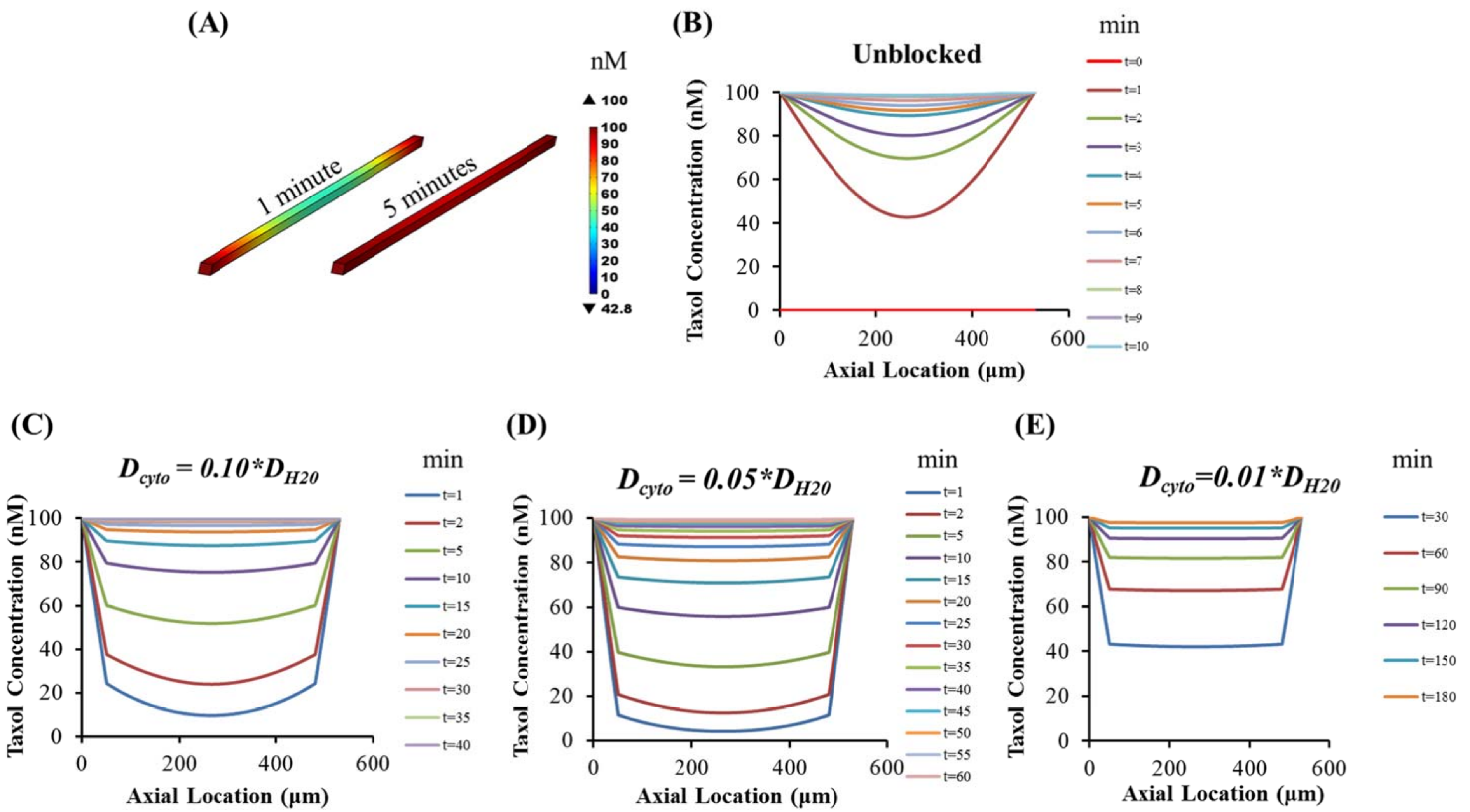

Figure S1. Taxol availability within the microchannel. (A) Volumetric Taxol concentration contours within an unblocked microchannel at t = 1 and 5 minutes. Simulation-predicted axial, centerline Taxol concentration profiles were illustrated in an unblocked microchannel (B), in a blocked microchannel where the Taxol diffusivity in cell D_{cyto} is (C) 10%, (D) 5% or (E) 1 % that in water, D_{H2O}. Time necessary for the concentration gradient to disappear ranged from 30 minutes to approximately less than three hours, indicated that steady state concentrations were achieved within three hours in the microchannels when D_{cyto} is at 1% of D_{H2O}.

Doxorubicin dose response

The dose response curves of *D54*, *D54-EGFRvIII* and GBM cells (Fig. S2A, B, and C) showed a plateau when Dox concentrations were below 10 nM. Linear responses occurred as Dox concentrations increased up to 1 μM. The estimated IC50 values were 120, 250, and 600 nM, respectively. In all cell lines, 1 μM Dox resulted in approximately less than 20% viability. As the resulting viability of cells in 2D was low, using Dox 1 μM enabled the investigation of any increasing viability in a confined environment.

Effect of low concentration Dox on the viability of GBM in physical confinement

Reduction of Dox concentrations from 1 μM to 100 nM and 500 nM resulted in overall higher viabilities (Fig. 6 and Fig. S2D). However, for both 100 nM and 500 nM, statistical analysis showed no significant difference of viabilities among three physical confinements, which was similar to the 1 μM case. These results reinforced our finding that Dox affected cancer cells regardless of their confinements, suggesting the need for the further investigation of effective therapeutic treatment on migrating cancer cells.

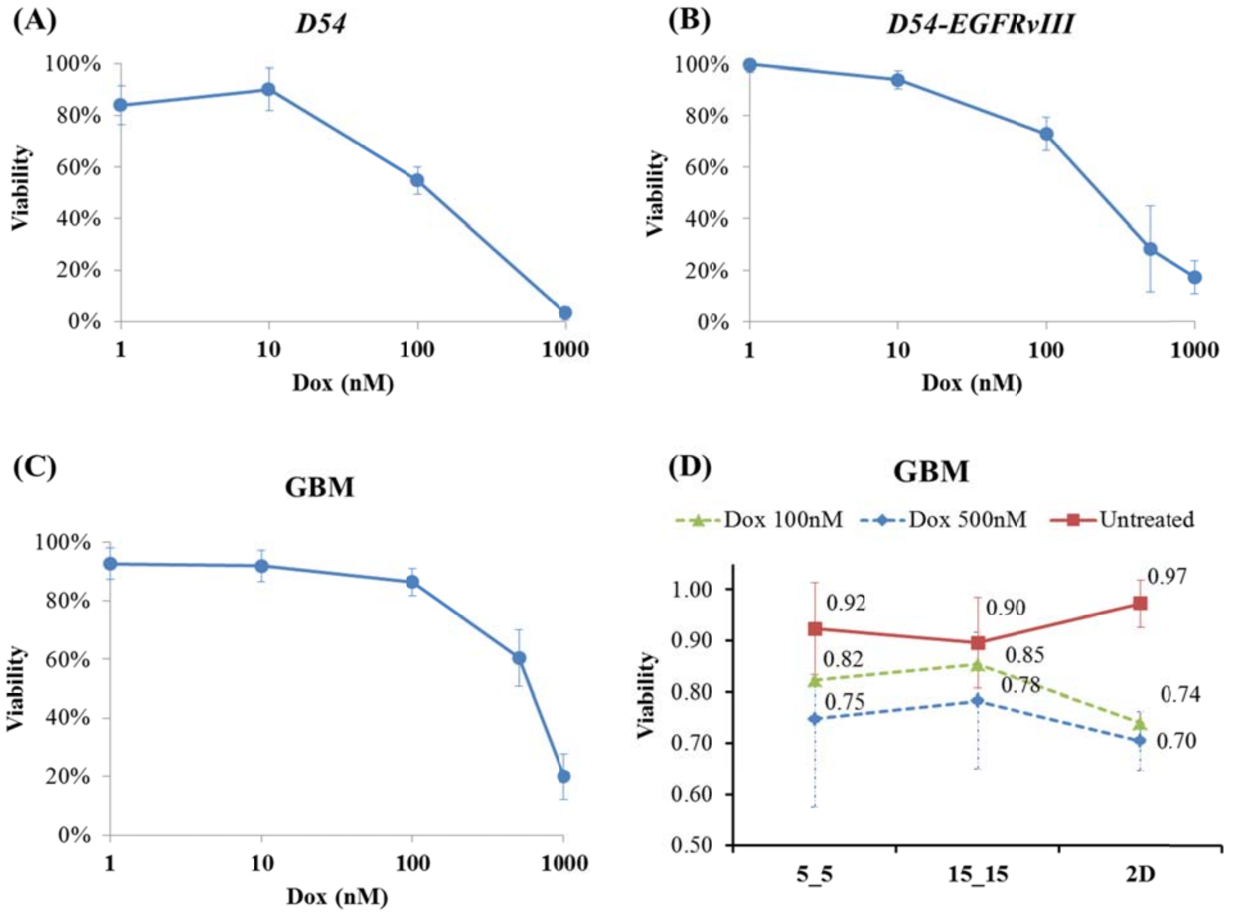

Figure S2. Doxorubicin dose response. (A, B, C) Dose response curves of *D54, D54-EGFRvIII* and GBM, respectively when cells grown on 2D were treated with different Dox concentrations for 48 hours. (D) Effect of lower Dox concentrations (100 nM green; 500 nM blue) on the viability of GBM in different physical confinements.

Supplementary References

S1. Cremasco, M.A.l. and L.N.-H. Wang, *Estimation of partition, free and specific diffusion coefficients of paclitaxel and taxanes in a fixed bed by moment analysis: experimental, modeling and simulation studies-doi: 10.4025/actascitechnol. v34i1. 8060.* Acta Scientiarum. Technology. **34**(1): p. 33-40
